# Will Gay Sex–Seeking Mobile Phone Applications Facilitate Group Sex? A Cross-Sectional Online Survey among Men Who Have Sex with Men in China

**DOI:** 10.1371/journal.pone.0167238

**Published:** 2016-11-23

**Authors:** Weiming Tang, Songyuan Tang, Yilu Qin, Ye Zhang, Wei Zhang, Chuncheng Liu, Lai Sze Tso, Chongyi Wei, Ligang Yang, Shujie Huang, Bin Yang, Joseph Tucker

**Affiliations:** 1 University of North Carolina Project-China, Guangzhou, China; 2 Guangdong Provincial Center for Skin Diseases and STI Control, Guangzhou, China; 3 SESH Global, Guangzhou, China; 4 School of Public Health, Kunming Medical University, Kunming, China; 5 Department of Epidemiology and Biostatistics & Global Health Sciences, University of California, San Francisco, California, United States of America; The First affiliated Hospital of China Medical University, CHINA

## Abstract

**Introduction:**

China is amidst a sexual revolution, with changing sexual practices and behaviors. Sex–seeking mobile phone applications (gay apps) that allow multiple people to meet up quickly may facilitate group sex. This study was therefore undertaken to evaluate group sex among Chinese MSM and to better understand factors associated with group sex.

**Methods:**

An online survey was conducted from September-October 2014, collecting data on socio-demographics, sexual behaviors, use of gay apps and occurrence of group sex among Chinese MSM. Univariate and multivariable logistic regressions were used to compare group sex and non-group sex participants.

**Results:**

Of the 1,424 MSM, the majority were under 30 years old (77.5%), unmarried (83.9%), and were gay apps users (57.9%). Overall, 141 (9.9%) participants engaged in group sex in the last 12 months. Multivariate analyses showed that men living with HIV, engaged in condomless anal intercourse with men, and used gay apps were more likely to engage in group sex, with adjusted ORs of 3.74 (95% CI 1.92–7.28), 2.88 (95% CI 2.00–4.16) and 1.46 (95% CI: 1.00–2.13), respectively. Among gay app users, the likelihood of group sex increases with the number of sex partners and the number of sex acts with partners met through a gay app.

**Conclusions:**

Chinese MSM who engage in group sex are also more likely to engage in other risky sexual behaviors, and gay app use may facilitate group sex. Further research is needed among MSM who engage in group sex in order to target interventions and surveillance.

## Introduction

China is amidst a sexual revolution. Rapid changes in sexual norms have led to the widespread adoption of more liberal attitudes towards sex and sexuality [1]. Mobile phone sex-seeking apps (gay apps) are increasingly used to find partners [2, 3]. This new technology could facilitate group sex among men who have sex with men (MSM). For example, Blued, the most popular gay app in China, was able to reach a user base of 27 million people in 2016, a mere three years after first launching [4].

Gay apps’ geospatial technology could facilitate gay app users to find multiple partners more quickly and much easier, which in turn could make it easier for groups of MSM to engage in group sex. Mobile apps might impact sexual behaviors among MSM, such as group sex. Group sex is defined by the presence of three or more persons having sex together at the same time [5]. Group sex participants have been shown to have an increased risk of drug use [6], condomless sex [7], and multiple sexual partners [8].

With the dramatic increase in usage of gay apps among MSM in China, recognizing its potential correlation with group sex is important, especially since Chinese gay app users tend to have more partners [2] and may thus tend to have group sex. These apps provide easy access to multiple partners and a conduit for quick meet ups, thus likely contributing to modern era of group sex behavior. Given the limited research on relationships between group sex and gay app use, particular gay app use patterns [9], more investigation is needed.

The purpose of this study was to gain a better understanding of group sex among Chinese MSM by examining correlates of group sex, particularly the correlation between group sex and gay app use.

## Methods

An online survey was conducted from September-October 2014 to assess risk behaviors of Chinese MSM. The survey was conducted on three different popular gay web portals in China (Northern China: http://www.danlan.org, Southern China: http://www.yntz.net, and Eastern China: http://www.jstz.org). These web portals serve as common entry points for a number of different services including news exchange, social networking, seeking sex partners, advertisement for gay-specific products, and research.

### Survey development and Recruitment

In order to develop the online survey, we first interviewed 20 MSM and other key stakeholders, to evaluate what questions are proper to ask through online survey. Based on interview discussions and a literature search, the survey instrument was developed (the literature search was separated into different sections, based on the measures of this study, which was described in detail at the “Measures” part). After development, the instrument was further reviewed by Chinese MSM who had previously participated in online surveys, leaders of local community-based organization (CBO) leaders, social media experts, physicians and public health experts [2, 10]. The revised survey instrument was then piloted among 144 MSM prior to the final survey. We used the checklist for reporting results of Internet e-surveys (CHERRIES) [11].

For participant recruitment, banner advisements were placed on the homepage of each web portal (Northern China: http://www.danlan.org, Southern China: http://www.yntz.net, and Eastern China: http://www.jstz.org). In addition, advertisements for the survey were sent to registered users of the three web portals. By clicking the banner, the participants were directly linked to the survey. Before taking the survey, participants had to first sign the online consent form. To meet the inclusion criteria, participants had to be born as biologically male, 16 years of age or older, have engaged in anal sex with man at any point, and be willing to provide their cell phone number. At the end of the survey, an incentive of $8 USD was given to each participant in the form of mobile pop-up.

### Measures

Socio-demographic information regarding year of birth, occupation (student or not), marital status (never married or ever married), education (senior high school or below, college or bachelors, or Masters or PhD), annual income (less than 3000 USD, 3000–6000 USD, 6001-9600USD, 9601–15000 USD, or more than 15000 USD) and residence (urban or rural) was collected from each participant. All participants were also asked about whether or not they had used gay mobile applications (gay app) to look for partners in the last six months (yes/no), what their sexual orientation (gay or bisexual), whether they disclosed their sexual orientation to others (yes/no), and what their currently self-identified gender was (man or transgender).

In addition, participants were asked questions about whether or not they currently had a primary partner (yes or no), what their sexual role usually was during anal sex (insertive, acceptive or no preference), whether they ever had vaginal or oral sex with women (yes/no), whether they had engaged in group sex in the last 12 months (yes/no), whether they had engaged in commercial sex in the last 12 months (yes/no), whether they had condomless sex with women in the last three months (yes or no), whether they had condomless sex with men in the last three months(yes/no), whether they ever drank alcohol during or prior to having sex in the last three months(yes/no), whether they ever exchanged sex for gifts or money(yes/no), whether they ever experienced intimate partner abuse(yes/no), and whether they ever used gay mobile applications (gay app) to look for partners in the last six months(yes/no). Participants were also asked whether they ever tested for HIV or STIs.

### Statistical Analysis

Descriptive analyses were performed to describe the socio-demographics, risk behaviors, and HIV/other STI testing history among both participants who engaged in group sex in the last 12 months (group sex group) and those who did not (non-group sex group). Univariate and multivariable logistic regressions were used to compare between the group sex group and the non-group sex group, while demographic characteristics, including age, residence, education, and income were adjusted for in the multivariate logistic regression models. We also analyzed the correlation between gay app use features and group sex among gay app users by using multivariate logistic regression.

### Ethical Statement

This study was approved by the ethics review committees in China (Guangdong Provincial Center for Skin Diseases and STI Control) and the United States (University of North Carolina at Chapel Hill and the University of California, San Francisco). We did not obtain informed consent from parents of minors (<18 years old) in the study, and the ethics committee approved the inclusion of minors (<18 years old) without parent/guardian consent.

## Results

### Socio-demographics and sex behaviors

Of the 5,339 clicks, 1,536 withdrew from the survey prior to reading the consent form and 656 were excluded for not signing the consent form. In addition, 1,328 did not meet eligibility requirements and 395 duplicates were excluded (by checking recorded phone numbers).[12] Of the 1424 participants, most were under 30 years old (77.5%, 1104), and 41.3% (588) were students. Over four-fifths (83.9%, 1194) of participants were unmarried. Around three quarters (74.1%, 1055) had at least college education, the majority had an annual income under $8000 USD (81.9%, 1166), and resided in an urban area (88.9%, 1266). In addition, about three-fifths (57.9%) of participants were gay apps users (Table 1).

**Table 1:** Socio-demographic characters and behaviors among Chinese MSM who engaged and not engaged in group sex, 2014 (n = 1424).

Variables		Group sex			Non-group sex			Total	
		Frequency	Percent	95% CI	Frequency	Percent	95% CI	Frequency	Percent
Age (years)	*Less than 20*	13	9.2	4.4,14.0	193	15	13.1,17.0	206	14.5
	*20 to 29*	81	57.4	49.2, 65.7	817	63.7	61.0,66.3	898	63.1
	*30 or above*	47	33.3	25.5,41.2	273	21.3	19.0,23.5	320	22.5
Education	*High school or less*	29	20.6	13.8,27.3	340	26.5	24.1,28.9	369	25.9
	*College / Bachelors*	94	66.7	58.8,74.5	875	68.2	65.6,70.8	969	68.1
	*Masters or PhD*	18	12.8	7.2,18.3	68	5.3	4.1,6.5	86	6
Student	*Yes*	45	31.9	24.1,39.7	543	42.3	39.6,45.0	588	41.3
	*No*	96	68.1	60.3,75.9	740	57.7	55.0,60.4	836	58.7
Marital Status	*Never married*	99	70.2	62.6,77.8	1095	85.3	83.4,87.3	1194	83.9
	*Ever Married*	42	29.8	22.2,37.4	188	14.7	12.7,16.6	230	16.1
Annual income (USD)	*<3000*	22	15.6	9.5,21.7	348	27.1	24.7,29.6	370	26
	*3000–6000*	31	22	15.1,28.9	389	30.3	27.8,32.8	420	29.5
	*6001–9600*	42	29.8	22.2,37.4	334	26	23.6,28.4	376	26.4
	*9601–15*,*000*	28	19.9	13.2,26.5	143	11.1	9.4,12.9	171	12
	*>15*,*000*	18	12.8	7.2,18.3	69	5.4	4.1,6.6	87	6.1
Residence*	*Urban*	129	91.5	86.8,96.2	1137	88.6	86.9,90.4	1266	88.9
	*Rural*	12	8.5	3.8,13.2	146	11.4	9.6,13.1	158	11.1
Gay app users^¶^	*Yes*	90	63.8	55.8,71.9	734	57.2	54.5,59.9	824	57.9
	*No*	51	36.2	28.1,44.2	549	42.8	40.1,45.5	600	42.1
Sexual Orientation	*Gay*	94	66.7	58.8,74.5	944	73.6	71.2,76.0	1038	72.9
	*Bisexual*	47	33.3	25.5,41.2	339	26.4	24.0,28.8	386	27.1
Sexual orientation disclosure	*Yes*	11	7.8	3.3,12.3	57	4.4	3.3,5.6	68	4.8
	*No*	130	92.2	87.7,96.7	1226	95.6	94.4,96.7	1356	95.2
Transgender Ф	*Yes*	16	11.3	6.0,16.6	45	3.5	2.5,4.5	61	4.3
	*No*	125	88.7	83.4,94.0	1238	96.5	95.5,97.5	1363	95.7
Currently have a main partner	*Yes*	91	64.5	56.6,72.5	600	46.8	44.0,49.5	691	48.5
	*No*	50	35.5	27.5,43.4	683	53.2	50.5,56.0	733	51.5
Main sexual role during anal sex	*Insertive*	72	51.1	42.7,59.4	452	35.2	32.6,37.8	524	36.8
	*Acceptive*	50	35.5	27.5,43.4	571	44.5	41.8,47.2	621	43.6
	*No preference*	19	13.5	7.8,19.2	260	20.3	18.1,22.5	279	19.6
Ever had vaginal or oral sex with women	*Yes*	80	56.7	48.5,65.0	334	26	23.6,28.4	414	29.1
	*No*	61	43.3	35.0,51.5	949	74	71.6,76.4	1010	70.9
Had condomless sex with women in the last 3 months	*Yes*	51	63.8	53.0,74.5	131	39.2	34.0,44.5	182	44
	*No*	29	36.3	25.5,47.0	203	60.8	55.5,66.0	232	56
Changed sex for gift/money in the last 12 months	*Yes*	35	24.8	17.6,32.0	47	3.7	2.6,4.7	82	5.8
	*No*	106	75.2	68.0,82.4	1236	96.3	95.3,97.4	1342	94.2
Intimate partner abuse^†^	*Yes*	104	73.8	66.4,81.1	676	52.7	50.0,55.4	780	54.8
	*No*	37	26.2	18.9,33.6	607	47.3	44.6,50.0	644	45.2
Had condomless sex with men in the last three months	*Yes*	66	46.8	38.5,55.2	937	73	70.6,75.5	1003	70.4
	*No*	75	53.2	44.8,61.5	346	27	24.5,29.4	421	29.6
Ever tested for HIV	*Yes*	91	64.5	56.6,72.5	612	47.7	45.0,50.4	703	49.4
	*No*	50	35.5	27.5,43.4	671	52.3	49.6,55.0	721	50.6
Ever tested for other STIs except HIV	*Yes*	75	53.2	44.8,61.5	381	29.7	27.3,32.2	456	32
	*No*	66	46.8	38.5,55.2	902	70.3	67.8,72.8	968	68

* Residence: the place the participants mainly live in during the survey time

^¶^ Gay app users: If participants reported any use of gay apps to find sex partners in the last six months, they were categorized as gay app users, while others were categorized as non-users

^Ф^ Transgender: Participants who have gender identities, expressions, or behaviors not traditionally associated with their birth sex

^†^Intimate partner: The term “intimate partner” includes current or former spouses, spouses in the process of separating, and dating partners.

Among survey respondents, 72.9% (1038) identified as gay and 27.1% (386) as bisexual. 29.6% (421) participants reported having engaged in condomless anal intercourse with men in the past three months. When engaging in anal intercourse, 36.8% (524) of participants preferred insertive sex, 43.6% (621) preferred receptive sex, and the rest 19.6% (279) had no preference. Moreover, 54.8% (780) of respondents reported that they had ever experienced intimate partner violence, and 5.8% (82) had exchanged sex for gifts or money in the last 12 months. Around half (49.6%) of respondents had ever tested for HIV, and one-third (32.0%) had tested for other STIs besides HIV.

### Factors Associated with Group Sex

Overall, 9.9% (141) of survey participants reported engaging in group sex in the prior 12 months.

Univariate analysis showed that MSM who were married were more likely to have engaged in group sex in the prior 12 months (crude OR = 2.47; 95% CI: 1.67, 3.66). Transgender individuals had a higher likelihood of engaging in group sex in the prior 12 months when compared to others (crude OR = 3.52; 95% CI: 1.93, 6.41). MSM who reported condomless sex with women in the last three months also had a higher likelihood of engaging in group sex (crude OR = 2.72; 95% CI: 1.64, 4.52). Similarly, MSM who had condomless sex with men within the past six months were more likely to have engaged in group sex in the prior 12 months (crude OR = 3.08; 95% CI: 2.16, 4.38), compared to MSM who consistently used condoms in the last three months. HIV testers were more likely to have engaged in group sex in the prior 12 months compared to MSM who never tested for HIV. Further, MSM with history of ever having a positive HIV result had a higher likelihood of engaging in group sex (crude OR = 3.80; 95% CI: 2.00, 7.21) (Table 2).

**Table 2:** Factors associate to group sex among Chinese MSM from an online survey, 2014 (N = 1424).

	Crude Model		Adjusted Model*	
Variables	OR (95% CI)	P-value	OR (95% CI)	P-value
**Marital status**				
*Never Married*	*Ref*		*Ref*	
*Ever married*	2.47(1.67, 3.66)	<0.001	1.72(1.07, 2.77)	0.025
**Ever tested for HIV**				
*NO*	*Ref*		*Ref*	
*Yes and HIV positive*	3.80(2.00,7.21)	<0.001	3.74(1.92,7.28)	<0.001
*Yes and HIV negative*	1.76(1.20,2.59)	0.004	1.51 (1.01,2.26)	0.046
*Yes but forgot results*	2.40(1.15,4.98)	0.019	2.22(1.04,4.75)	0.039
**Student**				
*No*	*Ref*		*Ref*	
*Yes*	0.64(0.44,0.93)	0.018	0.95(0.60,1.49)	0.82
**Transgender**				
*No*	*Ref*		*Ref*	
*Yes*	3.52(1.93,6.41)	<0.001	3.36(1.78,6.33)	<0.001
**Sexual orientation**				
*Bisexual*	*Ref*		*Ref*	
*Gay*	0.72(0.50,1.04)	0.081	0.82(0.55,1.21)	0.32
**Ever disclosed sexual orientation to others**				
*No*	*Ref*		*Ref*	
*Yes*	1.82(0.93,3.56)	0.080	1.56(0.78,3.14)	0.21
**Ever had sex with women**				
*No*	*Ref*		*Ref*	
*Yes*	3.73(2.61,5.32)	<0.001	3.09(2.03,4.72)	<0.001
**Had condomless sex with women in the last three months**				
*No*	*Ref*		*Ref*	
*Yes*	2.72(1.64,4.52)	<0.001	2.97(1.72,5.14)	<0.001
**Had condomless sex with men in the last three months**				
*No*	*Ref*		*Ref*	
*Yes*	3.08(2.16, 4.38)	<0.001	2.88 (2.00,4.16)	<0.001
**Ever had sex in exchange for gifts or money in the last 12 months**				
*No*	*Ref*		*Ref*	
*Yes*	8.68(5.37,14.04)	<0.001	10.81(6.41,18.25)	<0.001
**Ever tested for other STIs except HIV**				
*No*	*Ref*		*Ref*	
*Yes*	2.69(1.89,3.82)	<0.001	2.31(1.60,3.32)	<0.001
**Currently have a main sexual partner**				
*No*	*Ref*		*Ref*	
*Yes*	2.07(1.44,2.98)	<0.001	1.77(1.22,2.57)	0.003
**Ever experienced intimate partner abuse**				
*No*	*Ref*		*Ref*	
*Yes*	2.52(1.71,3.73)	<0.001	2.43(1.63,3.63)	<0.001
**Used gay apps for partners seeking in the last six months**				
*No*	*Ref*		*Ref*	
*Yes*	1.32(0.92,1.89)	0.13	1.46(1.00, 2.13)	0.051

* Model adjusted for age, residence, education, marital status and income.

After adjusting for age, residence, education, marital status and income, we found that group sex was associated with engaging in condomless anal intercourse with men in the last three months (OR 2.88, 95% CI 2.00–4.16), experiencing intimate partner violence (OR 2.43, 95% CI 1.63–3.63), being married (OR 1.72, 95% CI 1.07–2.77), and identifying as transgender (OR 3.36, 95% CI 1.78–6.33) (Table 2). Group sex was also positively associated with having a positive HIV test result (OR 3.74, 95% CI 1.92–7.28), a history of STI testing (OR 2.31, 95% CI 1.60–3.32), and exchanging sex for gifts or money in the last 12 months (OR 10.81, 95% CI 6.61–18.25). Meanwhile, we found that gay app users overall were more likely to engage in group sex with an adjusted OR of 1.46 (95% CI: 1.00–2.13).

### Correlation between gay app use features and group sex

In this study, we found that gay app users were more likely to engage in group sex with an adjusted OR of 1.46 (95% CI: 1.00–2.13). We therefore further analyzed the correlation between gay app use features and group sex among 824 gay app users. Multivariate regression analyses (adjusted for age, residence, education and income, Table 3) showed that the likelihood of group sex increases with the number of sex partners found through a gay app in the last 6 months, the number of receptive anal sex acts with partners met through a gay app in the last six months, and the number of insertive anal sex acts with partners; with adjusted ORs of 11.44 (95% CI: 5.21,25.13), 5.76 (95% CI: 2.66,12.47) and 16.24 (95% CI: 7.53, 34.98), respectively. Gay app users who had engaged in group sex during the prior 12 months were more likely to ask for the HIV status of their previous gay app partner before meeting in person (adjusted OR = 1.64, 95% CI: 1.03, 2.60).

**Table 3:** Correlation between gay app use features and group sex among Chinese MSM gay app users, 2014 (N = 824).

		Group sex	No group sex (n2)	Crude OR	95% CI	Adjusted OR^€^	95% CI
Time started to use gay app	*< 6 months*	19	118	0.66	0.33,1.34	0.93	0.44,1.97
	*6–12 months*	19	178	0.44	0.22,0.88	0.63	0.30,1.31
	*1–3 years*	34	364	0.38	0.21,0.72	0.49	0.26,0.93
	*> 3 years*	*18*	*74*	*Ref*		*Ref*	
Number of sex partners found through a gay app in the last 6 months	*1*	*9*	*215*	*Ref*		*Ref*	
	*2 to 3*	22	312	1.68	0.76,3.73	1.61	0.71,3.64
	*4 to 6*	15	107	3.35	1.42,7.90	3.01	1.24,7.30
	*Above six*	44	100	10.51	4.94,22.37	11.44	5.21,25.13
Number of receptive anal sex acts with partners met through a gay app in the last six months	*0 to 5*	*48*	*581*	*Ref*		*Ref*	
	*6 to 10*	17	90	2.29	1.26,4.15	2.70	1.44,5.06
	*11 to 20*	12	33	4.40	2.14,9.08	5.02	2.34,10.77
	*Above 20*	13	30	5.25	2.57,10.72	5.76	2.66,12.47
Number of insertive anal sex acts with partners met through a gay app in the last six months	*0 to 5*	*35*	*589*	*Ref*		*Ref*	
	*6 to 10*	19	109	2.88	1.59,5.23	2.57	1.39,4.75
	*11 to 20*	16	28	9.45	4.68,19.09	7.70	3.69,16.09
	*Above 20*	20	18	18.38	8.92,37.85	16.24	7.53,34.98
Time duration between met the last sex partner through a gay app and met in person	*< 1 hour*	10	65	1.72	0.75,3.94	1.46	0.66,3.22
	*1–24 hours*	37	188	2.20	1.20,4.04	0.68	0.30,1.53
	*2–7 days*	26	291	1.00	0.43,1.89	0.62	0.26,1.48
	*>1 week*	17	190	Ref		Ref	
Place had sex with the last gay app partner	*Home*	*29*	*346*	*Ref*		*Ref*	
	*Hotel or venues*	61	388	1.59	1.00,2.53	1.48	0.91,2.42
Used condom during sex with last gay app partner	*Yes*	*58*	*469*	*Ref*			
	*No*	*26*	*153*	*1*.*37*	*0*.*84*, *2*.*26*	*1*.*45*	*0*.*86*, *2*.*44*
Negotiated about condom use with the last gay app partner before met in person	*Yes*	*55*	*431*	*Ref*		*Ref*	
	*No*	35	303	0.90	0.58,1.42	0.94	0.59,1.50
Asked for HIV status of the last gay app partner before met	*No*	*50*	*500*	*Ref*		*Ref*	
	*Yes*	40	234	1.71	1.10,2.66	1.64	1.03,2.60

€ Model adjusted for age, residence, education, marital status and income.

## Discussion

Group sex often co-occurs with other high-risk behaviors that together create an environment conducive to disease transmission [6]. Group sex was more common among gay app users and men living with HIV. Our study expands on the existing literature on group sex [13, 14] by using online MSM portals, obtaining detailed information on gay apps, and exploring the correlation between group sex and gay app use. Our findings indicate that gay app use may create a virtual risk environment and facilitate group sex.

Nearly 10% of the MSM in our study reported engaging in group sex within the past 12 months, which is consistent with figures reported in previous studies conducted in China [13–15] as well as other low-and middle-income countries (LMICs) [16]. This frequency of group sex among MSM is lower than that reported in United States and Australia [9, 17]. This disparity may in part derive from differences the level of acceptance towards non-mainstream sexual identities and activities.

Our study found that group sex with associated with several risky sexual behaviors. This is consistent with research from the United States, Australia, and UK [16–18]. For instance, one study in Australia found that men who engaged in group sex during last six months were more likely to engage in unprotected anal intercourse. Another study in the US showed that the number of unprotected anal receptive sex acts at sex parties in the past 12 months was significantly associated with increased odds of engaging in unprotected anal sex with serodiscordant partners at the sex parties.

Our study found that MSM living with HIV were more likely to engage in group sex than other MSM. This is consistent with observations from Australia and the US [9, 17]. Our study did not collect targeted information about HIV serosorting. Nevertheless, our results are able to indicate a high prevalence of condomless group sex among MSM living with HIV, a concerning phenomenon with important public health implications.

Our results suggest that group sex is associated with gay app use. This finding is consistent with research from the United States [19, 20]. Gay apps have geospatial technology that could facilitate rapid identification sex partners and networking unbound by the constraints of time or location, facilitating rapid sex encounters. We found certain specific gay app features were particularly associated with a higher frequency of group sex. For instance, the more sex partners found through a gay app in the last 6 months, the more likely to engage in group sex. Gay apps’ geospatial technology facilitates rapid sex partner identification and networking regardless of time or location [7], which may also facilitate group sex among gay apps user.

We also found an association between group sex and having sex in exchange for gifts or money. Among the 82 MSM who reported having sex in exchange for gifts or money in our study, 32 (42.6%) reported having engaged in group sex. This finding is consistent with a study conducted in Bangladesh, which reported that group sex is common among male sex workers [21]. Our finding suggests that there is an intersection between group sex and commercial sex. We encourage future research that focuses on money boys or male sex workers to give heed to group sex behavior.

There were certain limitations with internal validity and external validity of this study. First, as this study was based on an online survey, selection bias may be an inherent part of the online recruitment approach, as it restricts those who were not able to access to Internet from participating. The MSM in our study were younger and more highly educated compared to non-online gay men [10]. However, national data [22] suggest that young gay men do carry a disproportionate burden of syphilis and HIV. A second limitation in our study is that all measured behaviors were based on self-report, and could thus be influenced by social desirability reporting bias. Third, a large number of MSM who clicked the survey link withdrew before being screened for eligibility, which may have resulted in selection bias. Therefore, the results need to be interpreted with caution. Finally, in the present study, we did not collect more detailed information regarding how group sex occurs and what type of condom behavior occurs during these encounters.

Despite these limitations, our study demonstrated that approximately one in ten MSM are engaging in group sex, a figure slightly lower than that found in Western countries but comparable to other low-and middle-income countries (LMICs). Group sex behavior may in fact be part of a syndemic[23] of interconnected high-risk health behaviors that co-occur and work synergistically to promote HIV transmission among MSM. These implications urge us to consider future interventions that pull together comprehensive resources to address the multiple manifestations of a complex problem. They represent a potential risk factor for the spread of HIV and syphilis that can be better targeted. Future studies might focus more on exploring and defining the specifics of group sex behavior, as well as associated high-risk activities such as drug use. Furthermore, insight from anthropological studies may shed light on motivators and drivers of group sex, as qualitative research has better capacity to explore social and psychological aspects of behavior. Gaining a better understanding of group sex is highly relevant from a public health and medical standpoint.
